# Cardiovascular system changes in rheumatoid arthritis patients with continued low disease activity

**DOI:** 10.1007/s00296-018-4053-x

**Published:** 2018-05-17

**Authors:** Małgorzata Biskup, Wojciech Biskup, Maria Majdan, Bożena Targońska-Stępniak

**Affiliations:** 1 Wojewódzki Zespół Specjalistyczny, Rzeszów, Poland; 2 Department of Rheumatology and Connective Tissue Diseases, Medical University of Lublin, Ul. Jaczewskiego 8, 20-950 Lublin, Poland

**Keywords:** Rheumatoid arthritis, Atherosclerosis, Carotid intima media thickness, Echocardiography, Diastolic dysfunction

## Abstract

Systemic inflammation and disease activity seem to contribute to excessive prevalence of cardiovascular (CV) diseases (CVDs) in patients with rheumatoid arthritis (RA). The objective of the study was to assess chosen CV parameters in RA patients who have continuous low disease activity. The study group consisted of 70 RA patients without known CVD and 33 healthy controls, of a comparable age. All RA patients had continued low disease activity (DAS28 ≤ 3.2) from 2 to 7 years. The groups were assessed for: blood pressure, serum amino-terminal pro-brain natriuretic peptide (NT-proBNP), carotid intima media thickness (cIMT), electrocardiography (ECG), ejection fraction (EJ) and diastolic dysfunction (*E*/*A* ratio) in echocardiography. In RA patients in comparison with controls, significantly greater values of cIMT [0.83 (0.21) vs 0.62 (0.1) mm, *p* < 0.001] were found, as well as higher incidence of atherosclerotic plaques [43 (61.4%) vs 10 (30.3%), *p* = 0.003], prolonged QTc interval [439.6 (23.7) vs 414.0 (27.9) ms, *p* < 0.001]. High or very high Systemic Coronary Risk Evaluation (SCORE) was found in 32.9% of patients with RA and increased serum NT-proBNP in 71.4%. The mean values of CV parameters (cIMT, E/A, NT-proBNP, SCORE) were associated with age, disease duration, rheumatoid factor (RF-IgM), erythrocyte sedimentation rate (ESR). The results of our study indicate, that RA with continued low disease activity is associated with atherosclerosis and heart dysfunction. Strong relationships were found between CV parameters and patients’ age, disease duration. Deterioration of CV parameters was associated with higher DAS28, ESR, RF-IgM concentration and bone erosions.

## Introduction

Rheumatoid arthritis (RA) is a chronic, immunologically mediated disease, manifesting as synovitis of multiple, peripheral joints and resulting in irreversible joint damage. The risk of death is twofold higher in RA patients than in general population and the main cause is cardiovascular disease (CVD), accounting for about a half of premature deaths observed. Traditional risk factors do not fully explain the increased CVD risk [1, 2]. Currently, RA is regarded as an independent risk factor for CVD, likewise type 2 diabetes [3]. According to literature, patients with RA and without clinically evident CVD, have higher prevalence of left ventricular (LV) diastolic dysfunction (DD) in comparison with controls [4].

High disease activity is reported to be associated with higher incidence of CVD, chronic heart failure (CHF) and mortality of patients with RA [5]. The increased CVD risk was found to be correlated with higher levels of anti-citrullinated protein/peptide antibodies (ACPA), elevated erythrocyte sedimentation rate (ESR) and C-reactive protein (CRP) both in RA patients and general population [6–9]. Active inflammation may also affect traditional CV risk factors, including hyperlipidaemia, obesity and insulin resistance [9].

In literature, there are few papers concerning CVD risk and heart function in patients with low disease activity, according to DAS28 (DAS28 ≤ 3.2) [10–13].

The aim of the study was to assess features of atherosclerosis and heart dysfunction in RA patients with continued low disease activity (DAS28 ≤ 3.2) and no history of CVD, using methods available on an outpatient basis.

## Materials and methods

The study was conducted on outpatients with RA, treated in the Regional Outpatient Clinic in Rzeszów, Poland. All patients fulfilled the American College of Rheumatology (ACR)/ European League Against Rheumatism (EULAR) classification criteria for RA [14]. The protocol of the study has been approved by the Ethics Committee of the Medical University of Lublin, with the approval number KE-0254/92/2013 and have therefore been performed in accordance with the ethical standards laid down in the 1964 Declaration of Helsinki and its later amendments. The informed consent was obtained from each patient, after an adequate explanation of design of the research.

The study group consisted of 70 RA patients who had continued low disease activity [4.2 (1.2) years, from 2 to 7 years] and no clinically evident CVD [e.g., ischemic heart diseases (IHD), CHF, hypertension], as well as other diseases which increase CV risk [diabetes, chronic kidney disease (CKD)]. The control group consisted of 33 healthy volunteers.

Demographic and clinical information was obtained through structured interview, review of medical records, self-report questionnaires, physical examination and laboratory tests. Assessment of CV system was performed using the following methods: electrocardiogram (ECG), echocardiography and high-resolution B-mode ultrasonography.

### RA-related data collection

Disease activity of RA was assessed using the Disease Activity Score (DAS) based on evaluation of 28 joints (DAS28), calculated with the number of tender and swollen joints, ESR value and patient’s global assessment of disease activity in visual analogue scale (VAS) [15]. The cut points of DAS28 are as follows: for low disease activity DAS28 ≤ 3.2, for remission < 2.6.

Erosive form of RA was diagnosed in those patients, who presented erosions on joint surfaces of bones in radiograms of hands and/or feet. Radiographs of hands and feet were made up to 6 months before study entry and assessed according to the Sharp/van der Heijde score (SHS) by a trained radiologist [16].

### Laboratory tests

Blood was collected after overnight fasting. All the tests were performed in the Medical Laboratory Diagnostics of the Regional Outpatient Clinic, according to standardized laboratory methods. The standard assessment included: complete blood cell count, ESR, serum concentration of CRP, creatinine, glucose, alanine transaminase (ALT), total cholesterol (TC), high-density lipoprotein (HDL) cholesterol (HDL-C), low-density lipoprotein (LDL) cholesterol (LDL-C), triglycerides (TG). Renal function was estimated by assessing serum creatinine concentration and creatinine-based estimated glomerular filtration rate (eGFR) calculated using Modification of Diet in Renal Disease (MDRD) formula [17]. The laboratory normal values of lipids are as follows: TC < 190 mg/dl, HDL-C ≥ 45 mg/dl in women and ≥ 40 mg/dl in men, LDL-C < 115 mg/dl, TG ≤ 150 mg/dl. Atherogenic index (AI) was calculated as ratio of TC and HDL-C concentrations with the normal value in women < 4.0 and in men < 4.5.

Blood samples were also taken to assess RA serological markers: IgM-rheumatoid factor (RF-IgM) and anti-citrullinated peptide (anti-CCP) antibodies, using enzyme-linked immunosorbent assays (ELISA). RF-IgM was determined using Johnson&Johnson ELISA assay, with the normal upper limit 12 units (IU)/ml. Anti-CCP antibodies were determined using the EUROIMMUN ELISA assay, with the normal upper limit 5 units (RU)/ml. Serum samples were also stored at − 80 °C for further assessment of amino-terminal pro-brain natriuretic peptide (NT-proBNP).

### CV parameters

Traditional and nontraditional CV risk factors were assessed in every patient. Information concerning CVD, diabetes, and CKD was taken from medical records.

During physical examination, blood pressure (BP) was assessed in a sitting position.

Height and weight were measured barefoot wearing light clothes. Body mass index (BMI) was calculated as the ratio of weight and squared height.

The 10-year risk of fatal CVD using the Systemic Coronary Risk Evaluation (SCORE) model was estimated in every patient, with the value > 5% indicating high risk. According to the EULAR recommendations, the result was multiplied by 1.5 (mSCORE) [18].

Measurement of NT-proBNP serum concentration was performed using electrochemiluminescence immunoassay (ECLIA), using Cobas assay (Elecsys proBNP II test). The recommended normal range is up to 125 pg/ml in patients < 75 year of age and up to 450 pg/ml in older ones.

### Electrocardiogram (ECG)

ECG was performed in RA patients and controls. Resting 12-lead ECGs (25 mm/s paper speed and 10 mm/mV amplitude) were recorded using a three-channel direct writing machine (ASPEL-Mr Gold). ECGs were reviewed by an independent cardiologist. For each ECG, data on following parameters were recorded: heart rate, QRS interval, QT interval, heart rate corrected QT (QTc) as calculated using the Bazett’s formula. According to American Heart Association/American College of Cardiology (AHA/ACC) guidelines, the normal QTc value is: < 450 ms in women and < 430 ms in men; the prolonged OTc is defined as: ≥ 460 ms in women and ≥ 450 ms in men [19].

### Echocardiographic examination

Echocardiographic examination was performed in RA patients and controls. All the assessments were performed by an independent cardiologist, unaware of the patients’ data, using a commercially available system (Philips HD15XE). Subjects were examined in left lateral recumbent position using standard parasternal views and apical views. Left ventricular (LV) end-systolic (LVESD) and end-diastolic (LVEDD) dimensions, LV wall thickness, left atrial diameter (LA) were measured by two-dimensional guided M mode echocardiography. LV function was assessed by ejection fraction (EF). Early (*E*) and late (*A*) diastolic mitral inflow velocities were measured by standard and tissue Doppler imaging (TDI) and *E*/*A* ratio calculated. The result of *E*/*A* ratio ≥ 1.0 is defined as normal and < 1.0 is equivalent for DD.

### Carotid intima media thickness (cIMT) measurement

An assessment of cIMT was performed in RA patients and controls. cIMT was measured using high-resolution B-mode ultrasound (Philips HD15XE). In every subject, IMT was assessed bilaterally in the three regions: common carotid artery (CCA), carotid bulb (BULB) and internal carotid artery (ICA). The average of maximum IMT from all 6 carotid segments (defined as mean cIMT) was used in the analyses. The mean cIMT value < 0.6 mm is considered as normal, ≥ 0.9 mm as abnormal. The cIMT value ≥ 0.6 and < 0.9 mm is a marker of subclinical atherosclerosis. The presence of carotid plaques is a marker of advanced atherosclerosis. Plaques were defined as a distinct protrusion, greater than 1.5 mm into the vessel lumen [20].

### Statistical analysis

Results were expressed as mean (standard deviation, SD) or number (%) and range of values (minimum and maximum). Variables were tested for normality by the Kolmogorov–Smirnov’s test. Differences between RA patients and controls as well as between the specific groups of RA patients (distribution of groups based on the disease duration, clinical and serological status) were tested using Kruskal–Wallis *H* test, Mann–Whitney *U* test as well as Student *t* test and Chi square test, for non-normally and normally distributed parameters, respectively. Spearman’s or Pearson’s correlation test was used to determine the association between the analyzed variables. Multivariable analysis (multiple linear regression) was performed according to a forward selection procedure, introducing those variables that showed a statistically significant association with the assessed parameters. For all tests, *p* values < 0.05 were considered significant.

## Results

### Demographic and disease-related variables in RA patients

A clinical characteristic of patients with RA has been presented in Table 1 and laboratory results in Table 2.

**Table 1:** Clinical characteristics of 70 RA patients

Variables	Results
Demographic variables	
Age, years, mean (SD) (range)	53.9 (13.1) (27–74)
Gender, F/M, *n* (%)	54 (77.1)/16 (22.9)
Cardiovascular risk factors	
Non-smokers, *n* (%)	63 (90.0)
Ex-smokers, *n* (%)	5 (7.1)
Current smokers, *n* (%)	2 (2.9)
BMI, kg/m^2^, mean (SD) (range)	25.2 (3.9) (17.0–39.1)
Normal BMI, *n* (%)	40 (57.1)
BMI > 30 kg/m^2^, *n* (%)	9 (12.9)
mSCORE, %, mean (SD) (range)	3.43 (3.19) (0–12)
Low risk, *n* (%)	17 (24.3)
High/very high risk, *n* (%)	23 (32.9)
RA-related variables	
Disease duration, years, mean (SD) (range)	6.9 (3.3) (3–16)
Erosions (hands/feet), *n* (%)	37 (52.9)
Extra-articular manifestations, *n* (%)	14 (22.9)
DAS28, mean (SD) (range)	2.87 (0.2) (2.7–3.2)
Treatment received	
Current glucocorticoid use, *n* (%)	16 (22.8)
Current conventional DMARD, *n* (%)	70 (100)
Current MTX monotherapy, *n* (%)	47 (67.1)
Current LEF monotherapy, *n* (%)	11 (15.7)
Current HCQ monotherapy, *n* (%)	5 (7.2)
Current SS monotherapy, *n* (%)	1 (1.4)
Current CsA monotherapy, *n* (%)	1 (1.4)
Current DMARDs combination, *n* (%)	5 (7.2)

Data are presented as mean (SD) (range) or number (%)

*BMI* body mass index, *CsA* cyclosporine A, *DAS28* disease activity score in 28 joints, *DMARD* disease-modifying anti-rheumatic drug, *HCQ* hydroxychloroquine, *LEF* leflunomide, *mSCORE* multiplied systemic coronary risk evaluation, *MTX* methotrexate, *SS* sulfasalazine

**Table 2:** Laboratory results in 70 RA patients

Variables	Results
Positive RF-IgM, *n* (%)	54 (77.1)
Positive anti-CCP, *n* (%)	53 (75.7)
CRP, mg/l, mean (SD) (range)	8.7 (12.9) (1–24)
ESR, mm/h, mean (SD) (range)	14.8 (10.2) (3–46)
Hemoglobin, g/dl, mean (SD) (range)	13.4 (1.1) (10.6–15.9)
Serum creatinine, mg/dl, mean (SD) (range)	0.77 (0.12) (0.5–1.1)
eGFR, ml/min/1.73 m^2^, mean (SD) (range)	87.3 (17.4) (56–127)
Serum glucose, mg/dl, mean (SD) (range)	91.9 (5.2) (82–109)
Total cholesterol, mg/dl, mean (SD) (range)	196.7 (38.9) (125–312)
Normal total cholesterol, *n* (%)	36 (51.4)
HDL-cholesterol, mg/dl, mean (SD) (range)	57.0 (14.5) (31–90)
Normal HDL-cholesterol, *n* (%)	57 (81.4)
LDL-cholesterol, mg/dl, mean (SD) (range)	116.8 (35.7) (60–234)
Normal LDL-cholesterol, *n* (%)	37 (52.9)
Triglycerides, mg/dl, mean (SD) (range)	113.6 (55.4) (49–419)
Normal triglycerides, *n* (%)	56 (80.0)
AI, mean (SD) (range)	3.6 (1.2) (1.6–7.3)
Normal AI, *n* (%)	51 (72.9)
NT-proBNP, pg/ml, mean (SD) (range)	97.6 (63.4) (14.4–298.7)
Normal NT-proBNP (≤ 125 pg/ml), *n* (%)	50 (71.4)

Data are presented as mean (SD) (range) or number (%)

*AI* atherogenic index, *anti-CCP* anti-cyclic citrullinated peptide antibodies, *CRP* C-reactive protein, *eGFR* estimated glomerular filtration rate, *ESR* erythrocyte sedimentation rate, *NT-proBNP* amino-terminal pro-brain natriuretic peptide, *RF-IgM* IgM rheumatoid factor

The disease activity was low (DAS28 ≤ 3.2) in all RA patients. Most patients had erosive form of RA and over ¾ of them were seropositive (RF-IgM and/or ACPA). Extra-articular manifestations (rheumatoid nodules) were found in 14 (22.9%) patients.

At the time of examination, conventional synthetic disease modifying anti-rheumatic drugs (csDMARDs) were used in all patients and included: methotrexate (MTX) in almost ¾ of patients (dose 10–25 mg/week, in monotherapy or combination), leflunomide (LEF), hydroxychloroquine (HCQ), sulfasalazine (SS) and cyclosporine A (CsA). Therapy with low-dose glucocorticoid (GC) (prednisone ≤ 5 mg/day) was used in 16 patients (Table 1). There were no patients treated with biological DMARDs (bDMARDs).

Patients with RA included in the study had no history of IHD, CHF, hypertension, diabetes, CKD. A majority of them were non-smokers, with normal AI and BMI values. NT-proBNP concentration remained within the referenced ranges in almost ¾ of patients (Table 2).

According to the mSCORE system, low risk of 10-year CV death was found in ¼ of patients. High or very high risk was noticed in 1/3 of patients with RA and no CVD history (Table 1).

### Characteristics of the control group

The control group consisted of 33 healthy subjects: 18 women (54.5%) and 15 men (45.5%) with the mean (SD) age of 53.6 (8.3) years (range 27–75). They had no traditional CV risk factors.

### Comparison of CV parameters in groups of patients and controls

The comparison of CV parameters has been presented in Table 3. The mean age did not differ significantly between patients and controls.

**Table 3:** Comparison of CV parameters in RA patients and controls

Variables	Patients	Controls	*p*
Age, years, mean (SD) (range)	53.9 (13.1) (27–74)	53.6 (8.3) (27–75)	NS
SBP, mmHg, mean (SD) (range)	128.3 (9.4) (110–145)	131.3 (9.6) (120–150)	NS
DBP, mmHg, mean (SD) (range)	81.0 (7.4) (70–95)	82.7 (5.2) (70–95)	NS
*E*/*A* ratio, mean (SD) (range)	1.08 (0.28) (0.56–1.9)	0.99 (0.21) (0.6–1.3)	NS
Abnormal *E*/*A, n* (%)	26 (37.1)	10 (30.3)	NS
EF, %, mean (SD) (range)	59.8 (1.6) (50–65)	60.2 (0.9) (60–65)	NS
QTc, ms, mean (SD) (range)	439.6 (23.7) (384–515)	414.0 (27.9) (374–509)	< 0.001
cIMT, mm, mean (SD) (range)	0.83 (0.21) (0.33–1.37)	0.62 (0.1) (0.45–0.85)	< 0.001
Normal cIMT (< 0.6 mm), *n* (%)	10 (14.3)	12 (36.4)	*p* = 0.01
Arterial wall hypertrophy (cIMT > 0.9 mm), *n* (%)	26 (37.1)	0	*p* = 0.0001
Carotid plaques presence, *n* (%)	43 (61.4)	10 (30.3)	*p* = 0.003

Data are presented as mean (SD) (range) or number (%)

*cIMT* carotid intima media thickness, *DBP* diastolic blood pressure, *E*/*A ratio* early/late mitral inflow velocities, *EF* ejection fraction, *SBP* systolic blood pressure

The mean value of cIMT was significantly higher in RA patients than in controls (Table 3). The incidence of normal cIMT value (< 0.6 mm) was significantly lower in RA patients than in controls, in spite of comparable age of both groups. The incidence of atherosclerotic plaques and arterial wall hypertrophy (cIMT > 0.9 mm), indicating advanced atherosclerosis was found significantly more often in RA patients than in controls (Table 3).

The value of QTc was significantly higher in RA patients than in controls (25-ms) (Table 3).

There were no statistically significant differences between patients and controls in respect to values of SBP, DBP, *E*/*A* ratio and EF.

### Correlations between CV parameters and RA disease markers

We noted strong correlations between CV parameters and both patients’ age and disease duration (positive with mean values of cIMT, mSCORE and NT-proBNP, as well as negative with *E*/*A* ratio) (Table 4). The mean values of *E*/*A* ratio, mSCORE and NT-proBNP concentration correlated with the mean ESR value (Table 4). There were positive associations between NT-proBNP and disease activity expressed as DAS28, as well as between mSCORE and RF-IgM concentration (Table 4).

**Table 4:** Correlations between CV parameters and RA disease activity characteristics

Variable	cIMT		*E/A* ratio		NT-proBNP		mSCORE	
	*R*	*p*	*R*	*p*	*R*	*p*	*R*	*p*
Age	0.52	< 0.001	− 0.55	< 0.001	0.37	0.002	0.83	< 0.001
RA duration	0.42	< 0.001	− 0.27	0.02	0.33	0.006	0.23	0.05
CRP	0.08	NS	− 0.11	NS	0.1	NS	0.03	NS
ESR	0.12	NS	− 0.3	0.01	0.33	0.005	0.3	0.01
DAS28	0.17	NS	− 0.21	NS	0.25	0.04	0.18	NS
RF-IgM	− 0.08	NS	− 0.21	NS	0.17	NS	0.25	0.04
Anti-CCP	0.01	NS	− 0.09	NS	0.02	NS	0.12	NS

*Anti-CCP* anti-cyclic citrullinated peptide antibodies, *cIMT* carotid intima media thickness, *CRP* C-reactive protein, *DAS28* Disease Activity Score in 28 joints, *E*/*A ratio* early/late mitral inflow velocities, *EF* ejection fraction, *ESR* erythrocyte sedimentation rate, *NT-proBNP* amino-terminal pro-brain natriuretic peptide, *RF-IgM* IgM rheumatoid factor, *mSCORE* multiplied systemic coronary risk evaluation

In the multiple linear regression analysis, significant associations were confirmed for: cIMT with age (*b* = 0.007, *p* = 0.0002) and disease duration (*b* = 0.02, *p* = 0.002); *E*/*A* ratio with age (*b* = − 0.01, *p* = 0.002); NT-proBNP with ESR (*b* = 2.1, *p* = 0.02) and disease duration (*b* = 6.4, *p* = 0.002); mSCORE with RF-IgM (*b* = 0.007, *p* = 0.0009) and age (*b* = 0.2, *p* < 0.0001).

No correlation was found between CV parameters and mean concentrations of CRP, anti-CCP. We found no correlation between mean values of EF or QTc and RA disease markers.

### Characteristics of RA patients with and without atherosclerosis

**Fig. 1:**
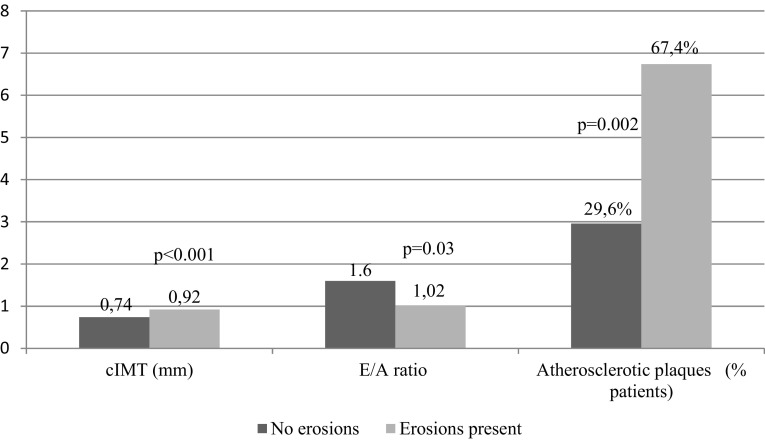
Comparison of cardiovascular parameters in RA patients with no erosions vs erosions present

Patients with abnormal cIMT (≥ 0.6 mm) were characterized by higher disease activity parameters (ESR, DAS28) and were significantly older than patients with normal cIMT (< 0.6 mm) (Table 5). The mean cIMT value was significantly higher in patients with bone erosions compared without erosions (*p* < 0.001) (Fig. 1).

**Table 5:** Differences of RA disease parameters in patients with normal and abnormal values of cIMT and *E*/*A* ratio

Variables	cIMT			*E*/*A* ratio		
	Normal	Increased	*p*	Normal	Decreased	*p*
Age	38.7 (9.2)	56.3 (11.9)	< 0.001	48.5 (12.7)	62.8 (7.9)	< 0.001
RA duration	5.3 (1.1)	7.3 (3.4)	NS	6.2 (2.8)	7.9 (3.7)	0.03
ESR	8.9 (3.7)	15.7 (10.5)	< 0.05	12 (7.5)	19.3 (12.3)	0.003
DAS28	2.75 (0.17)	2.89 (0.2)	< 0.05	2.83 (0.19)	2.93 (0.2)	< 0.05
RF-IgM	75.9 (46.3)	73.9 (110.9)	NS	51.8 (48.0)	112.1 (153.2)	0.02

Data are presented as mean (SD)

*cIMT* carotid intima media thickness, *DAS28* disease activity score in 28 joints, *E*/*A ratio* early/late mitral inflow velocities, *ESR* erythrocyte sedimentation rate, *RF-IgM* IgM rheumatoid factor

Higher values of ESR and age were noted in patients with atherosclerotic plaques. Significantly higher incidence of bone erosions was found in patients with atherosclerotic plaques compared without plaques (*p* = 0.002) (Fig. 1).

### Characteristics of RA patients with *E*/*A* ratio: normal (≥ 1) vs abnormal (< 1)

Patients with abnormal *E*/*A* ratio value were characterized by higher inflammatory and immunological parameters (ESR, DAS28, RF-IgM concentration) as well as longer disease duration and age (Table 5).

The mean value of *E*/*A* was significantly lower in patients with bone erosions compared without erosions (*p* = 0.03) (Fig. 1).

## Discussion

In our study, the higher burden of atherosclerosis, revealed by higher cIMT value and presence of plaques, was found in RA patients in comparison with healthy controls. Significantly longer QTc in RA patients might suggest higher risk of sudden cardiac death (SCD). It is noteworthy, that in patients with continued low disease activity and no history of CVD, high or very high risk of CV death according to mSCORE, was noted in 1/3 of patients and NT-proBNP was elevated above the normal range in almost 1/4. These results suggest higher cardiovascular risk in patients with low RA activity in comparison with healthy controls.

Strong relationships were noted between CV parameters, disease duration and patients’ age, which is also expected in the general population. Simultaneously, significant correlations between CV parameters and the disease activity parameters suggest substantial impact of inflammatory activity on atherosclerosis and heart dysfunction in RA patients.

RA is currently regarded as a novel, independent CVD risk factor. Accelerated atherosclerosis, considered as an extra-articular manifestation of RA, occurs as a result of interaction between traditional CV risk factors and inflammatory activity of joint disease. Both atherosclerosis and RA have in common inflammatory mediators. The mechanisms which induce synovial inflammation are similar to those in unstable atherosclerotic plaque [1, 21].

According to literature, age has been reported as the major determinant of subclinical atherosclerosis in patients with different types of arthritis; contributions of other traditional CV risk factors and disease activity to be limited [1]. The results of our study also point to a significant relationship between age and both atherosclerosis and heart dysfunction. Despite comparable age of patients and healthy controls, intensity of atherosclerosis was significantly higher in RA patients, suggesting additional impact of inflammation.

The beneficial effect of low disease activity has been suggested in RA patients. It has been reported, that atherosclerosis is not accelerated in RA of low activity or remission [11] and low disease activity is sufficient to achieve a protective effect against CVD in RA [13]. Remission defined as DAS28 < 2.6 has no additional protective effect against CVD compared with low disease activity [13]. Moreover, in RA patients with remission/low disease activity, atherosclerosis indices are comparable between patients treated with bDMARDs and treated with csDMARDs [11]. In the group of RA patients with low disease activity (median DAS28 2.4), cIMT was not increased compared to controls and cIMT was not associated with RA disease characteristics. In RA patients, hypertension and age were associated with cIMT, in controls—age and smoking. The authors concluded, that treatment of hypertension and other traditional CVD risk factors seem warranted to reduce CV risk in RA [12]. Conversely, results of another study showed that, in patients with inactive RA cIMT value was significantly higher and atherosclerotic plaques were more frequent than in controls [10]. In our study, we observed acceleration of atherosclerosis in RA patients compared with controls. Amongst patients with continued low disease activity, those with lower activity parameters (ESR and DAS28) had no features of atherosclerosis.

The prolongation of QTc interval is an established electrocardiographic predictor of the risk of arrhythmia and sudden death in the general population. It was reported, that a 50-ms increase of QTc was associated with doubling of the risk of all-cause mortality. Contemporary association was observed between QTc and CRP levels, suggesting that the prolonged QTc interval was driven by a high inflammatory burden [22, 23]. We found the significantly higher mean QTc interval in RA patients than in controls, without relationship with the current disease activity markers.

The growing evidence from observational studies suggests that DMARDs, in particular MTX and biologics, are associated with decreased CV mortality and morbidity. However, there are no studies specifically evaluating the impact of csDMARDS and few studies evaluating the impact of bDMARDs on SCD, incidence of arrhythmias or QTc interval in RA patients [24, 25]. DMARDs are expected to exert the beneficial effect directly due to reduction in inflammation and as a result reduction in CVD burden. In the study of 17 patients with active RA, 76% of which had a prolonged QTc, it was found, that treatment with bDMARD (tocilizumab) was associated with rapid and significant reduction in QTc duration and correlated with the decrease in both CRP and TNF-α concentrations [25]. Similar results, reduction in both QTc interval and inflammatory markers, were found in patients with ankylosing spondylitis, following treatment with infliximab [26]. However, the use of biologics (particularly anti-TNF antibodies) is not without the risk. In literature, case reports described severe arrhythmias following biological treatment [24].

Patients with RA have approximately twofold higher incidence of CHF, which is usually clinically silent and more likely with a preserved EF. An association of high ESR and RF positivity with LV dysfunction was reported in RA [27]. Isolated DD may be one mechanism for the excess development of CHF [4, 28], manifested among others by lower *E*/*A* ratio [29]. An aberrant immune response was considered as a contributing factor to DD development in RA patients. It was reported, that disease duration and interleukin-6 (IL-6) levels were significantly associated with DD [4]. Another study demonstrated, that patients with active RA have lower LV systolic myocardial function (diagnosed with speckle tracking echocardiography, STE) compared with patients in remission, independent of LV-EF, which could be associated with subclinical coronary artery disease [30]. The results of this study showed not significant differences of *E*/*A* ratio and EF between RA patients and controls. However, decreased *E*/*A* ratio (< 1) was observed in patients with higher disease activity markers (ESR, DAS28) and higher RF-IgM concentration.

Concentration of NT-proBNP was reported to be increased in patients with RA, independently of CV risk factors. An association was noted between NT-proBNP and CRP, supporting the link between myocardial dysfunction and inflammation [5]. In our study, NT-proBNP concentration was significantly correlated with ESR value and disease duration.

The results of this study indicate, that in patients with low disease activity, the burden of CV risk is associated with higher activity parameters (DAS28, ESR), RF-IgM concentration and bone damage.

The strengths of the presented study include: the homogeneous study population which consisted of patients with continued low disease activity and simultaneously no history of CVD or other diseases which increase CV risk; all the patients were treated with csDMARDs and there were no patients with biological treatment; all assessments performed are available on outpatient basis.

The limitations of the study include: higher number of patients could enable more accurate statistical analysis; the use of other, non-standard inflammatory markers could allow more precise assessment of inflammatory state in patients with low activity according to DAS28; ultrasound examination of joints could provide additional data to assess disease activity adequately; it would be rational to compare groups of patients with diverse inflammatory or autoimmune rheumatic diseases, due to observed enhanced atherosclerosis [31].

In conclusion, features of accelerated atherosclerosis and heart dysfunction were found in the group of RA patients with continued low disease activity (DAS28 ≤ 3.2). Strong relationship was noted between CV parameters, patients’ age and disease duration. Deterioration of CV parameters was observed in patients with higher DAS28, ESR, RF-IgM concentration and bone erosions.
